# An unusual pattern of three major components of the cardiovascular system: multimodality imaging and review of the literature

**DOI:** 10.1186/1749-8090-8-61

**Published:** 2013-04-04

**Authors:** Dimitris Flessas, Ioannis Mamarelis, Vasilis Maniatis, George Souretis, Nikolaos Laschos, Christophoros Kotoulas, Kyriakos Lazaridis

**Affiliations:** 1 Department of Cardiology, 417 VA Hospital (NIMTS), 10-12 Monis Petraki st, Athens, Greece; 2 Iaso General Hospital, Department of Cardiothoracic Surgery, 264 Mesogion Ave, Athens, Greece; 3 Iaso General Hospital, Department of Radiology, 264 Mesogion Ave, Athens, Greece

**Keywords:** Anomalous origin of coronary artery, Single coronary artery, Common origin from right sinus of Valsalva, Anomalous origin of the left common carotid artery, Aneurysm of the descending thoracic aorta

## Abstract

**Introduction:**

Coronary artery anomalies are found in 0.4% to 1.4% of patients who undergo coronary angiography. Anomalous origin of left coronary artery from the right sinus of Valsava is the rarest, with a reported prevalence of 0.02 –0.03% according to angiographic studies.

**Case presentation:**

We present the rare case of a 42-year-old-man suffering from stable angina with unusual development of 3 major components of the cardiovascular system Coronary angiography revealed an anomalous origin of the left coronary artery from the ostium of the right coronary artery. Magnetic resonance angiography depicted an anomalous origin of the left common carotid artery from the innominate artery and an aneurysm of descending thoracic aorta. Coronary computed tomography angiography revealed the course of left coronary artery between aortic root and conus arteriosus at the level of the right ventricular outflow tract. In this report we attempt to highlight the rarity of this vascular anatomy.

**Conclusion:**

Anomalous origins of the coronary arteries are rare, but may cause myocardial ischemia and sudden death. Thus, their reliable identification is a matter of paramount importance possibly evaluating the effects of therapeutic intervention. Newer imaging modalities improve the illumination of vascular system anatomy, shedding light to diagnostic dilemmas that come up in daily medical practice.

## Background

Coronary artery anomalies are found in 0.4% to 1.4% of patients who undergo coronary angiography. Increasing rates of intervention & surgery make this more clinically significant. Our case report describes an anomalous origin of the left coronary artery from the ostium of the right coronary artery, an ascending thoracic aorta aneurysm and an anomalous origin of the left common carotid artery from the proximal part of the brachiocephalic trunk, indentified during investigation of stable angina. Whilst most cases of coronary anomalies appear benign, there are specific rare patterns with malignant nature and with increased risk of ischemia and sudden cardiac death. Anomalous origin of a coronary artery from the opposite side of Valsava (ACAOS) with interarterial course (course between aorta and pulmonary artery) is a clinical entity with increased incidence for sudden cardiac death.

## Case presentation

We report an unusual pattern of 3 major components of the cardiovascular system in a 42-year-old Caucasian man who attended our hospital to undertake invasive coronary evaluation due to symptoms of stable angina. He had a history of arterial hypertension, dyslipidemia and a family history of coronary artery disease. Prior to admission, he presented in outpatient clinic with stable angina over the last two months, without ischaemic changes in rest electrocardiogram. Without receiving medication, he underwent a treadmill exercise test which was suspicious for ischaemic changes. Specifically the patient described chest pain of mild intensity at peak exercise with mild ST depression in the anterolateral leads.

**Figure 1:**
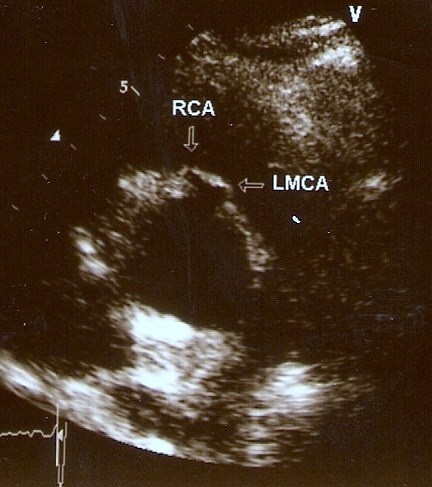
Common origin of both RCA and LMS from the right coronary cusp in echocardiogram.

**Figure 2:**
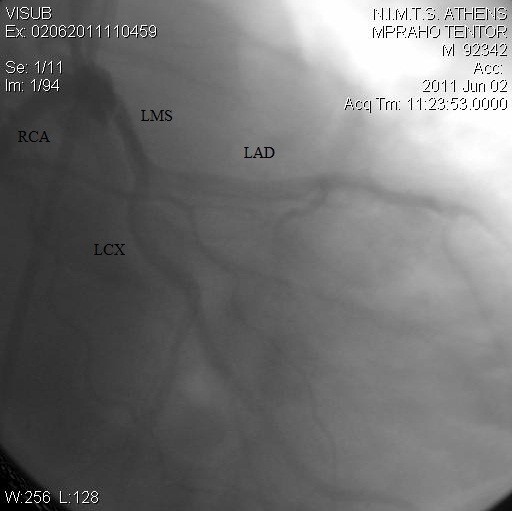
Common origin of both RCA and LMS from the right coronary cusp in the LAO view.

**Figure 3:**
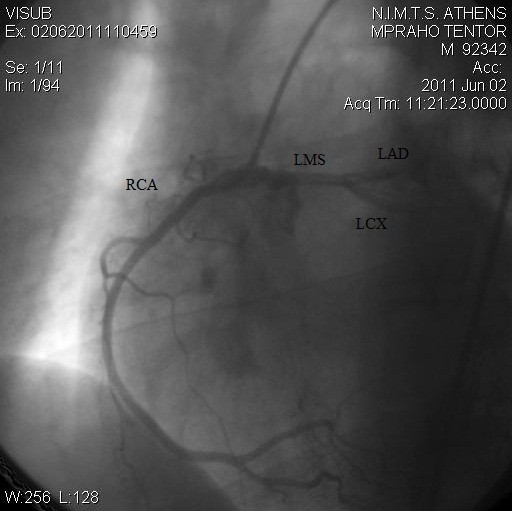
Common origin of both RCA and LMS from the right coronary cusp in the RAO caudal view.

A two-dimensional transthoracic echocardiogram (TTE) was performed. On short parasternal axis view at the level of great vessels the LCA seemed to share a common ostium with the RCA, originating from the right sinus of Valsava. No wall motion abnormalities were revealed, systolic function was normal and color-Doppler yielded no-pathologic findings [Figure 1]. The pericardium was free of fluid. The patient then underwent a coronary arteriogram which revealed no apparent coronary obstructive lesions. What was noteworthy was the anomalous origin of the left coronary artery from the right coronary sinus. The RCA shared a common ostium with the anomalous left coronary artery. In the right anterior oblique (RAO) projection, LCA -prior to its bifurcation- follows a cranial and leftward direction [Figures 2 and 3].

**Figure 4:**
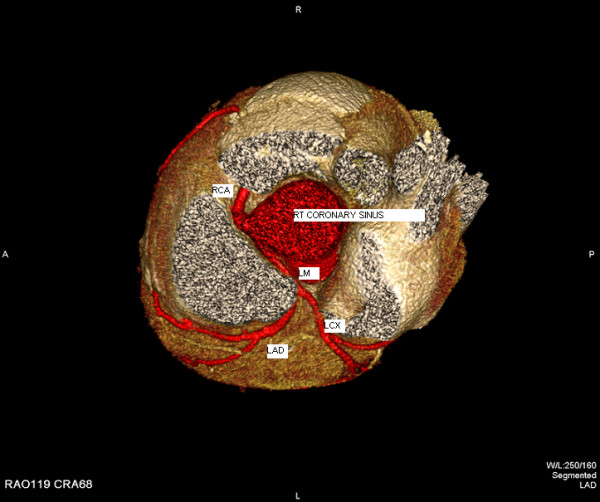
**3-D reconstruction of coronary arteries colored in red.** Common origin of LM and RCA and course of LCA.

**Figure 5:**
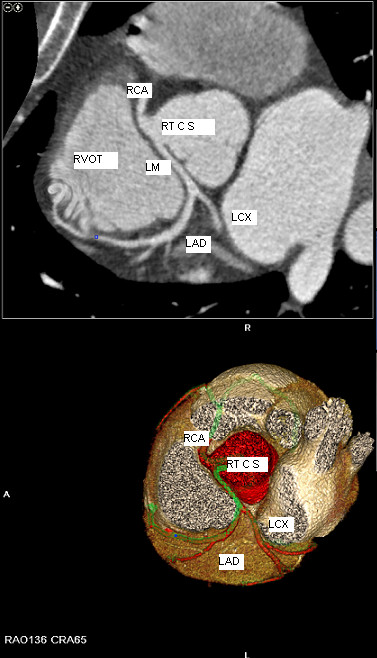
Oblique MIP of CCTA.

**Figure 6:**
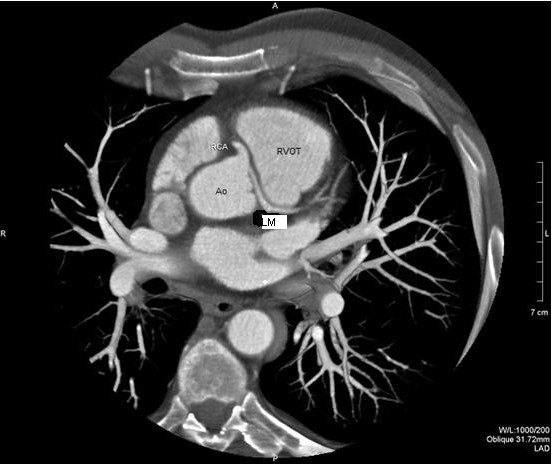
Oblique-axial MIP clearly marking the common origin of LM and RCA and the course of the LCA between the RVOT and the aortic root.

**Figure 7:**
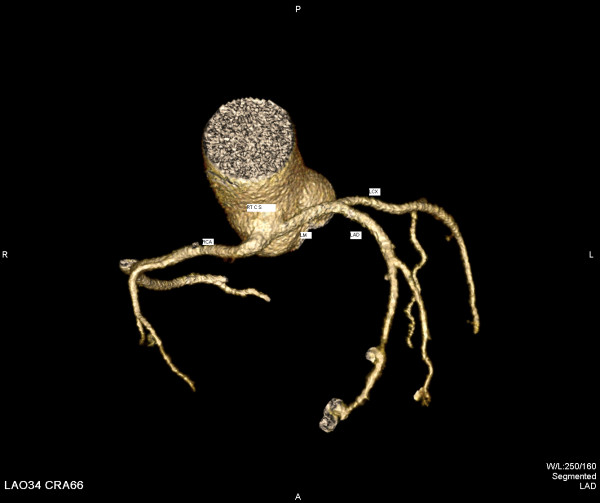
3-D reconstruction of CCTA – Vessels only projection.

**Figure 8:**
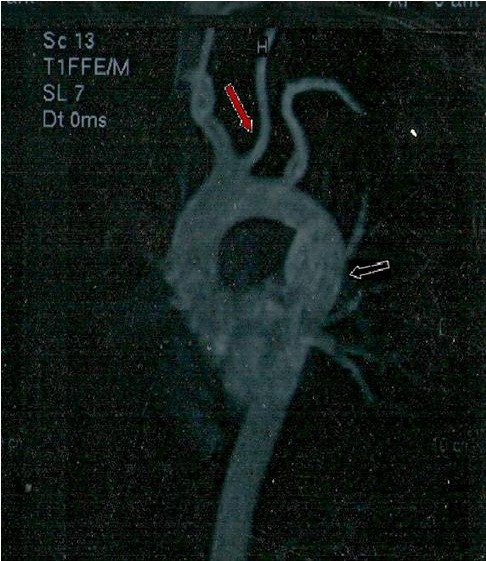
Anomalous origin of the left common carotid artery from the innominate artery (red arrow) and aneurysm of the descending thoracic aorta (white arrow).

**Figure 9:**
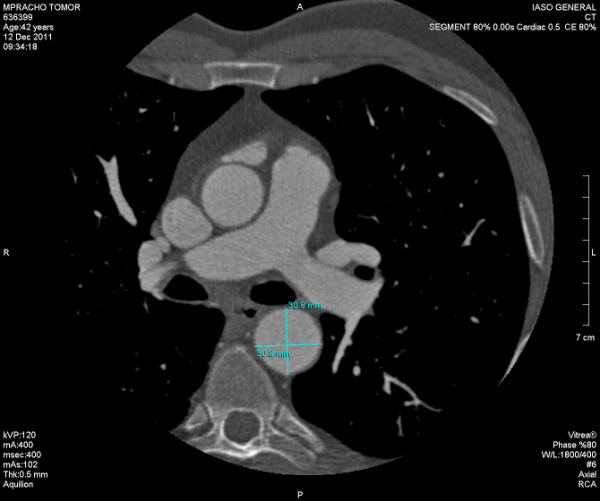
Aneurysm of the descending thoracic aorta with a transverse diameter of 3,1 cm.

In order to better deliniate the origin and the course of the coronary arteries our patient was referred for magnetic resonance angiography (MRA) and coronary computed tomography angiography (CCTA). CCTA findings [Figures 4, 5, 6, 7] were compatible with that of a congenital anomaly in the origin of the left coronary artery which originates from right Valsava sinus with a common ostium with the right coronary artery. Left main coronary artery is 2 cm in length (longer than average) follows an interarterial course, passing between aortic root and conus arteriosus at the level of the pulmonary valve. Immediately after its exit (between the aortic root and the right ventricular infundibulum), it bifurcates to LAD and LCx. The right coronary artery originates from the RSV, coursing between the root of the aorta and the right ventricular outflow tract. LAD in its mid portion presents myocardial bridging of non significant length and depth. Coronary vessels were without any critical stenosis and coronary artery calcium score was negative. MRA clearly depicted anomalous origin of the left common carotid artery from the proximal part of the brachiocephalic trunk (innominate artery) [Figure 8] and an aneurysm of the descending thoracic aorta with a transverse diameter of 3,1 cm [Figure 9] thus bringing the number of incidentally found concurrent abnormal arterial patterns to three.

Finally in order to rule out ischemia as a cause of angina patient was scheduled for SPECT. SPECT was negative. Angina was attributed neither to the coronary artery ectopy nor to the myocardial bridging.

## Discussion

Coronary anomalies affect less than 1% of the general population. Congenital anomalies of coronary arteries are a rare clinical entity, with the incidence report between 0.4–1.4% in angiographic series and 0.3% in autopsy series. Isolated single coronary artery is a very rare anomaly, with an incidence of 0.04% to 0.23% [1]. Anomalous origin of LCA from the RSV is the rarest, with a reported prevalence of 0.02–0.03% according to angiographic studies [1]. Congenital coronary artery anomalies are generally incidental, and asymptomatic, however some can cause severe potentially life threatening symptoms. Understanding coronary variations is the key in determining anomalous patterns associated with sudden cardiac death [2]. And understanding these conditions is becoming incresingly important with the recognition that anomalous coronary arteries are involved in 12% of sports-related sudden cardiac deaths versus 1.2% non-sports related deaths [2].

Single coronary artery has been defined angiographically by Lipton, Yamanaka and Hobbs. The latter modified the Lipton classification including features such as ostial location, anatomical distribution, and the course of the transverse trunk [3] [Table 1].

**Table 1:** Angiographic types of isolated single coronary artery (modified Lipton classification)

	**Code**	**Description**
Ostial location	R	Right Sinus of Valsava
	L	Left Sinus of Valsava
Anatomical distribution	I	The solitary dominant vessel follows the course of either a normal right or left coronary artery
	II	One coronary artery arises from the proximal part of the normally located another coronary artery
	III	LAD and LCx arise separately from a common trunk originating from the right sinus of Valsava
Course of the transverse trunk	A	Anterior to the great vessels
	B	Between the aorta and the pulmonary Arteries
	P	Posterior to the great vessels
	S	“Septal type” : A part of the route passes through the interventricular septum
	C	“Combined type” : Combination of diverse routes

Anomalous origin of a coronary artery from the opposite side of Valsava comprises a subset of coronary anomalies that may have severe prognostic implications. The interarterial course (vessel passes between aortic root and conus arteriosus) commonly occurs and is associated with sudden cardiac death. Anomalous origin of the LCA from the right sinus of Valsalva is consistently related to sudden death in 59% of patients. The course of anomalous artery between two great vessels is associated with acute myocardial infarction and sudden cardiac death [4]. The acute angle of the ostium and the compression between the commissure of the right and left coronary cusps increase the risk of sudden cardiac death. Exercise leads to expansion of the aortic root and pulmonary trunk, and may increase the existing angulation of the coronary artery, decreasing the luminal diameter in the proximal portion of the coronary artery. Resting ECGs are usually normal and stress tests are not always positive.

**Figure 10:**
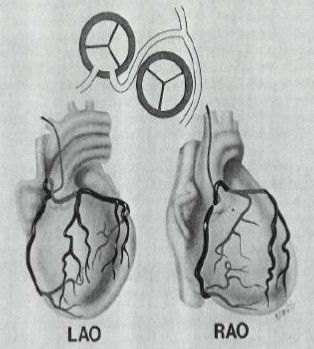
Graphical representation of LMCA from RSV and interarterial course.

As one can notice from the graphical representations of the coronary arteries [Figure 10], the entire LCA arises from the RSV. The RCA may arise separately or share a common ostium with the anomalous left coronary and is considered a form of single coronary artery. The LCA courses between aorta and pulmonary artery [5]. The difference of the anatomic variant [Figure 10] when compared with the normal anatomy is obvious and suggests its clinical significance.

The anomaly described in our patient appears potentially malignant, but without obvious compression between the great arteries. Currently only in a small, specific subgroup of left ACAOS cases –those that have an anomalous origin of the ectopic coronary artery within the wall of the aortic root with intussusceptions- are recognized as candidates for intervention [6]. In such cases, if associated with exertional angina or exertional syncope, then many cardiothoracic surgeons will offer re-implantation since coronary angioplasty is technically difficult [6].

The management of patients with an anomalous coronary artery includes medical treatment or observation, coronary angioplasty with stent deployment or surgical repair. The therapy of the anomalously originating LCA from the RSOV is mainly based on observations and experts opinion, since the literature consists of case reports [7]. There are two surgical approaches that can be used for treatment of ACAOS of the LCA. One is the direct repair of the anomalous origin in the aortic root, and the other is coronary artery bypass surgery. Direct repair of the anomalous proximal coronary segment has been reported, unroofing of the whole intussuscepted segment or the creation of new ostium at the distal end of that segment. Coronary artery bypass surgery is technically more feasible, but there are disadvantages with the grafts. Arterial grafts tend to atrophy or fail to develop when used to bypass coronary lesions that are not severely obstructive at baseline. Vein grafts have limitations on longevity. Percutaneous transluminal coronary angioplasty is technically difficult, and there are only a few reports of PTCA of anomalous coronary arteries including rotational atherectomy and stenting and laser angioplasty [8].

Although cardiac catheterization is recognized as the gold standard for the evaluation of coronary anomalies, CCTA has recently emerged as an effective noninvasive method to image the origin and course of the coronary arteries and to display the anatomy and assessment of cardiac structures. Particularly, the 3-dimentional nature and high spatial resolution of CCTA allow for the unambiguous determination of the origin and proximal course of anomalous coronary vessels in relation to surrounding cardiovascular structures as the aorta and the pulmonary artery [9].

Aneurysms of the supra-aortic vessels represent an unusual form of aneurysmal disease. Usually detected as asymptomatic masses, such aneurysms may cause life-threatening complications. Left common artery arising from the proximal part of the innominate artery appears with an incidence of 0.2% in the literature [10]. An association between left common artery arising from the initial part of the innominate artery (bovine arch variation) and aortic dilation is seen in older patients, when dilation involves the aortic arch. Bovine arch should be considered a potential risk factor for thoracic aortic aneurysm.

## Conclusion

The identification of the anomalous patterns of our cardiovascular system constitutes a matter of great clinical importance due it its fatal complications. The newer modalities that have emerged have enabled the accurate visualization of the anatomical configurations and the detection of the anatomic malformations, thus assisting us in performing precise evaluation and avoiding subsequent errors in management. Congenital coronary artery anomalies, until recently rarely identified during life, are closely associated to sudden cardiac death but are amenable to cardiac surgery. A variety of structural cardiovascular abnormalities generally incidental and asymptomatic, have been implicated as a cause of death among young people especially athletes, raising questions regarding the preventive clinical strategies and the need of an international registry of prospectively identified young patients with coronary artery anomalies. The subject of coronary artery anomalies is currently undergoing profound changes related to clinical presentation, diagnostic work up, prognosis and treatment.

## Consent

Written informed consent was obtained from the patient for publication of this Case report and any accompanying images. A copy of the written consent is available for review by the Editor-in-Chief of this journal.

## Abbreviations

CCTA: Coronary computed tomography angiography; RCA: Right coronary artery; LM: Left main coronary artery; LCX: Left circumflex artery; LAD: Left anterior descending artery; RT CS: Right coronary sinus; RVOT: Right ventricular outflow tract; ACAOS: Anomalous origin of a coronary artery from the opposite side of Valsava

## Competing interests

The authors declare that they have no competing interests.

## Authors’ contributions

DF wrote the manuscript, assisted in literature research and submitted the manuscript. IM performed the coronary angiography, conceived the idea and obtained the pictures. GM performed the CCTA and the MRA obtaining the pictures. GS and NL assisted in the literature research and further contributed to the manuscript. CK provided cardiothoracic consultation, assisted in reading and correcting the final manuscript. KL read and approved the final manuscript. All authors read and approved the final manuscript.
